# P10 Impact of miniaturization and early readout on antimicrobial susceptibility tests

**DOI:** 10.1093/jacamr/dlad066.014

**Published:** 2023-06-14

**Authors:** Sarah Needs, Jessica Hayward, Stephen Kidd, Al Edwards

**Affiliations:** School of Pharmacy, University of Reading, UK; School of Pharmacy, University of Reading, UK; Basingstoke and North Hampshire Hospital, Hampshire Hospitals NHS Foundation Trust, UK; School of Pharmacy, University of Reading, UK

## Abstract

**Background:**

New technologies offer the promise of faster, more accessible antibiotic susceptibility testing. UK policy includes the development and introduction of innovative diagnostics to tackle antimicrobial resistance, and whilst many new technologies are emerging, we still have limited evidence of their performance. Many of these emerging technologies are changing two core aspects of antimicrobial susceptibility tests (AST). Firstly, using microfluidic platforms to miniaturize standard antibiotic susceptibility testing and secondly measuring susceptibility at earlier timepoints, here, we report a comprehensive assessment of 1-mL volume broth microdilution with an early endpoint readout, proving that miniaturization and earlier timepoints of conventional antibiotic susceptibility testing is feasible and accurate.

**Methods:**

The test measures bacterial growth in 1-mL capillaries in the presence of the metabolic dye resazurin. All other aspects of the test remain standard. Images of the capillaries are taken every 5 minutes over 18 h to determine accuracy at different timepoints during incubation at 37°C. Over a total of 3 sites in the UK, Spain and Thailand, we have tested 367 isolates from clinical samples for 8–17 antibiotics.

**Results:**

Preliminary data shows that for 99 Gram-negative isolates tested in Thailand, after 6-h incubation, accuracy for antibiotics is as follows: cefotaxime 96%, ceftazidime 92%, meropenem 93%, amikacin 94%, amoxicillin–clavulanic acid 79%, cefuroxime 97%, cefoxitin 80%, ciprofloxacin 84%, gentamicin 93% and trimethoprim 97%. Further testing is ongoing, and isolates from urine samples are currently being collected from Basingstoke and North Hampshire hospital to expand the type of organism tested.

**Conclusions:**

Unlike many microfluidic systems, the dip and test microcapillary–based broth microdilution test is manufactured in bulk, allowing rapid and testing of large sample sets with minimal investment. An understanding of the effect of miniaturization is vital to developers of microfluidic phenotypic antibiotic susceptibility tests and can provide confidence in emerging systems.

